# Correction to “Nanoindentation study of human fingernail for determining its structural elasticity”

**DOI:** 10.1111/srt.13852

**Published:** 2024-07-17

Tohmyoh H, Abukawa M. Nanoindentation study of human fingernail for determining its structural elasticity. *Skin Res Technol*. 2023;29:e13456. doi:10.1111/srt.13456

In page 2, Equation (3) “ $R_{B}=\sum_{i=1}^{3}E_{i}I_{i}=\sum_{i=1}^{3}E_{i}\int_{l_{i}}^{u_{i}}{\left(y-\delta h\right)}^{2}bdy$” was incorrect. The correct equation is “ $R_{B}=\sum_{i=1}^{3}E_{i}I_{i}=\sum_{i=1}^{3}E_{i}\int_{l_{i}}^{u_{i}}{\left(y-\eta h\right)}^{2}bdy$”.

In page 3, Equation (4) “$\mathrm{SEB}\;=\frac{R_{B}}{I}=4E_{1}\left\{{\left[\kappa -(\frac{1}{2}+\eta )\right]}^{3}-{\left[-(\frac{1}{2}+\eta )\right]}^{3}\right\}+4E_{2}\left\{{\left[\lambda -(\frac{1}{2}+\eta )\right]}^{3}+{\left[\kappa -(\frac{1}{2}+\eta )\right]}^{3}\right\}+4E_{3}\left\{{\left[1-(\frac{1}{2}+\eta )\right]}^{3}+{\left[\lambda -(\frac{1}{2}+\eta )\right]}^{3}\right\}$” was incorrect. The correct equation is “$\mathrm{SEB}\;=\frac{R_{B}}{I}=4E_{1}\left\{{\left[\kappa -(\frac{1}{2}+\eta )\right]}^{3}-{\left[-(\frac{1}{2}+\eta )\right]}^{3}\right\}+4E_{2}\left\{{\left[\lambda -(\frac{1}{2}+\eta )\right]}^{3}-{\left[\kappa -(\frac{1}{2}+\eta )\right]}^{3}\right\}+4E_{3}\left\{{\left[1-(\frac{1}{2}+\eta )\right]}^{3}-{\left[\lambda -(\frac{1}{2}+\eta )\right]}^{3}\right\}$”.

We apologize for this error.

